# Investigating the incidence, nature, severity and potential causality of medication errors in hospital settings in Qatar

**DOI:** 10.1007/s11096-020-01108-y

**Published:** 2020-08-07

**Authors:** Binny Thomas, Abdulrouf Pallivalapila, Wessam El Kassem, Moza Al Hail, Vibhu Paudyal, James McLay, Katie MacLure, Derek Stewart

**Affiliations:** 1 Women’s Wellness and Research Center, Hamad Medical Corporation, Doha, Qatar; 2 Corporate Pharmacy Executive Office, Hamad Medical Corporation, Doha, Qatar; 3 School of Pharmacy, University of Birmingham, Birmingham, UK; 4 Institute of Medical Sciences, University of Aberdeen, Aberdeen, UK; 5 School of Pharmacy and Life Sciences, Robert Gordon University, Aberdeen, UK; 6 College of Pharmacy, QU Health, Qatar University, Doha, Qatar

**Keywords:** Causes, Error reporting, Incidence, Medication errors, Qatar, Reason’s accident causation model, Severity

## Abstract

*Background* Medication errors are a major public health concern that negatively impact patient safety and health outcomes. Effective and efficient medication error reporting systems and practices are imperative in reducing error incidence and severity. *Objective* The objectives were to quantify the incidence, nature and severity of medication errors, and to explore potential causality using a theoretical framework. *Setting* The study was conducted at Hamad Medical Corporation, the largest public funded academic healthcare center in the state of Qatar. *Methods* A retrospective review of medication error reports submitted to the Hamad Medical Corporation incident reporting system during 2015 to 2017. Data related to number of reports, reporter, medication, severity and outcomes were extracted. Reason’s Accident Causation Model was used as a theoretical framework for identifying potential causality. Two researchers independently categorized errors as: active failures (e.g. forgetting to administer medication at scheduled time); error provoking conditions (e.g. medication prescribed by an unauthorized physician and administered to the patient); and latent failures (e.g. organizational factors, lack of resources). *Main outcome measures* Incidence, classes of medications, reporter, error severity and outcomes, potential causality. *Results* A total of 5103 reports provided sufficient information to be included in the study giving an estimated error incidence of 0.044% of prescribed medication items. Most of the reports (91.5%, n = 4667) were submitted by pharmacists and majority (87.9%, n = 4485) were prescribing errors. The most commonly reported medications were anti-infectives for systemic use (22.0%, n = 1123) followed by medications to treat nervous system disorders (17.2%, n = 876). Only three errors reported to have caused temporary harm requiring intervention while one contributed to or resulted in temporary harm requiring initial or prolonged hospitalization. In terms of potential causality of medication errors, the majority (91.5%, n = 4671) were classified as active failures. *Conclusion* Almost all reports were submitted by pharmacists, indicating likely under-reporting affecting the actual incidence. Effort is required to increase the effectiveness and efficiency of the reporting system. The use of the theoretical framework allowed identification of potential causality, largely in relation to active failures, which can inform the basis of interventions to improve medication safety.

## Impacts on practice

- There is a need to improve the information supplied by healthcare professionals when submitting an error report.
- Accumulation of evidence around error causality using Reason’s Accident Causation theory will be useful in considering any potential interventions aiming to reduce these factors.
- Identifying behavioural determinants in relation to error reporting will help establish a better error reporting system.

## Introduction

Medication errors are highly prevalent, occurring at any stage of the medication use processes including prescribing, dispensing, medication administration and monitoring [1–5]. In March 2017, the World Health Organization (WHO) launched the third global patient safety challenge, ‘Medication Without Harm’ [6], following on from the first two challenges of, ‘Clean Care is Safer Care’ [7], and ‘Safe Surgery Saves Lives’ [8]. The third challenge aims to, ‘drive a process of change to reduce patient harm generated by unsafe medication practices and medication errors’. Medication errors are preventable, as highlighted in the most commonly cited definition of the National Coordinating Council for Medication Error Reporting and Prevention (NCCMERP) in the United States, ‘any preventable event that may cause or lead to inappropriate medication use or patient harm, while the medication is in the control of the health care professional, patient, or consumer’ [9].

A number of systematic and narrative reviews have sought to quantify mediation errors and the subcategories of prescribing, administration and dispensing errors [3, 10]. Ross et al. aimed to quantify prescribing errors committed by junior doctors, reporting that errors occurred at a rate of 2 to 514 per 1000 items prescribed and 4.2–82.0% of patient charts [11]. The systematic review of Lewis et al. on the prevalence, incidence and nature of prescribing errors in hospital inpatients yielded a median error rate [interquartile range (IQR)] of 7% (2–14%) of medication orders, 52 (8–227) errors per 100 admissions and 24 (6–212) errors per 1000 patient days [2]. Focusing on medication administration errors, Keers et al. reported a median error rate of 18.8% (IQR 4.9–23.5%) including with timing errors and 7.4% (5.2–9.8%) without [12]. Several systematic reviews have focused on studies conducted in the Middle East. In 2013, Alsulami et al. reviewed the incidence, types of medication errors and main contributory factors in Middle Eastern countries. While noting that error rates were difficult to compare due to being expressed differently, prescribing errors ranged from 7.1% of prescriptions in a teaching hospital to 90.5% of prescriptions in a primary healthcare centre. Poor medication knowledge was identified as a major contributory factor for errors [3]. In 2018, Alsaidan et al. reviewed studies which originated in the six countries of the Gulf Cooperation Council (Bahrain, Kuwait, Oman, Qatar, Saudi Arabia, The United Arab Emirates) giving a prescribing error rate of 8.5–16.9/100 admissions [13]. Later, Thomas et al. synthesized data on the prevalence, nature, severity and causes of medication errors in hospital settings in the Middle East. Prescribing errors were the most commonly studied, with a median error rate of 10% (IQR 2–35%). The review further reported that causes of medication errors were multifactorial and that a standardised, theory-informed approach should be adopted in primary research to ensure that all possible explanations underlying medication errors could be identified [10].

Reason’s Accident Causation model is the most commonly applied theoretical framework in identifying and addressing medication errors [14]. This model categorises error causes as active failures, error-producing conditions and latent failures. Active failures are unsafe acts committed by those in direct contact with the patient or system. These failures take a variety of forms including slips and lapses (errors in task execution), mistakes (errors in planning) and procedural violations (rule breaking). Error-producing conditions include those within the local workplace (e.g. time pressure, understaffing, inadequate equipment, fatigue and inexperience). Latent failures arise from decisions made by policy makers, leaders and top-level management [15]. Review of errors within these categories should stimulate reflection on practice at the individual and organisational levels in an attempt to eliminate further similar errors.

Effective and efficient medication error reporting systems are therefore of paramount importance to promote staff engagement; quality, timely and consistent reporting; and feedback to impact organisations and practitioners, and thus enhance patient safety [9, 16]. The strategic aims of NCCMERP highlight the value of effective and efficient medication error reporting systems and practices in reducing error prevalence and severity [9]. Goals include: stimulating the development and use of medication error reporting systems by healthcare organisations,and stimulating the review and analysis of error reports leading to the development of recommendations to reduce, and ultimately prevent, errors.

## Aim of the study

The aims of the study were to quantify the incidence, nature and severity of medication errors reported within Hamad Medical Corporation (HMC), Qatar, and to explore potential causality using Reason’s Accident Causation model.

### Ethics approval

The study was approved in September 2018 by the Institutional Ethics Committee and Medical Research Center at HMC (MRC-01-18-109).

## Methods

This was a cross-sectional, retrospective chart review study of all medication errors submitted by health professionals during the period of January 2015 to December 2017 (i.e. 36 months).

### Setting

This study was conducted at the largest academic healthcare center in the state of Qatar. With more than 2300 inpatient beds, HMC is the primary provider of secondary and tertiary healthcare in the country. HMC manages 12 hospitals, (nine specialist hospitals and three community hospitals), the National Ambulance Service and home and residential care services [17].

### Medication error reporting at HMC

HMC’s medication error reporting policy has adopted the NCCMERP definition of medication error, with health professionals mandated to report all medication errors and near misses, irrespective of severity or consequence [18]. A ‘near miss’ is defined within the policy as, ‘an event or situation that could have resulted in an accident, injury, or illness, but did not, either by chance or through timely intervention’. HMC advocates reporting all types of errors, and the importance of medication error reporting has been explained to all staff during orientation and also during periodic education and training in the form of lectures, seminars and conferences etc.

**Fig. 1:**
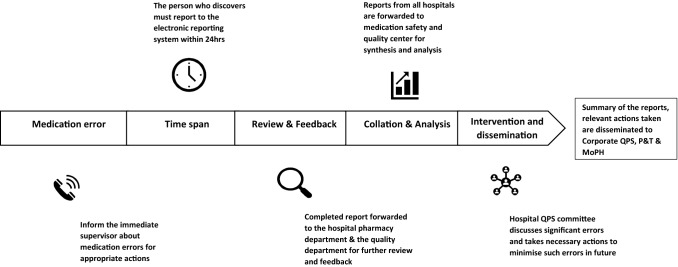
Medication error reporting process at Hamad Medical Corporation, Qatar

The medication error reporting system is electronic, with all reports being reviewed by the HMC Quality Management Department. Quarterly and annual reports on medication errors and near misses, including action taken, are shared with HMC Quality and Patient Safety Committee, and HMC Pharmacy and Therapeutic Committee [18]. The medication error reporting process is described in Fig. 1.

### RL6 incident reporting system

RL6 is a web-based online reporting system adapted by HMC for voluntarily reporting of medication errors by health professionals using a standardized format. An electronic form captures details of, reporter; date and time of error; area (inpatient/outpatient); hospital; patient details; staff involved; description of the error; medications involved; type of error (prescribing, dispensing etc.); severity of error; and contributory factors [19].

### Analysis

All reports were extracted from the RL6 database using IBM SPSS Statistics for Windows, Version 20.0. Data cleaning was performed, and multiple reports of the same event were counted as one; if the same error was reported by multiple health professionals, only the first report was included.

The incidence of medication errors was calculated using the formula,

$${\text{Incidence}} = \frac{{\text{Total number of medication errors reported}}}{{\text{Total number of medications ordered}}} \times 100\%$$

The total number of medications ordered over the study period was generated by Cerner, an internationally accepted health information technology used in more than 27,000 healthcare facility across the world. It is an integrated database that electronically stores, captures and accesses patient health information. One medication ‘order’ represents each medication item prescribed to an individual patient, irrespective of dose, route, duration etc. The following were data also extracted: the specific medication categorized according to the WHO Anatomical Therapeutic Chemical (ATC) classification [20], profession of the reporter,and severity assessed by the reporter using the NCCMERP approach of classification into four levels and nine severity categories ranging from potential for error (category A) to actual error that may have contributed to or resulted in a patient’s death (category I) [21]. These data were analysed using descriptive statistics of frequency and percentage.

The free text data on contributory factors (potential causality) of medication errors recorded by the reporter were independently analysed by two researchers experienced in the assessment of medication error reports. Instances of non-consensus were referred to one of two other experienced assessors for final judgement. Each researcher applied Reason’s Accident Causation Model as a framework [15], categorizing potential causality as: active failures,error provoking conditions,or latent failures. These were sub-classified by the researchers according to the most commonly recurring groupings.

## Results

Over the three-year study period, 18,390 incidents were reported, of which 2130 were excluded as duplicates and a further 2720 excluded as not deemed errors (e.g. out of stock medication and non-preventable adverse drug reactions). During the same period, there were 30,650,000 medication orders giving a reported medication error incidence of 0.044%.

**Fig. 2:**
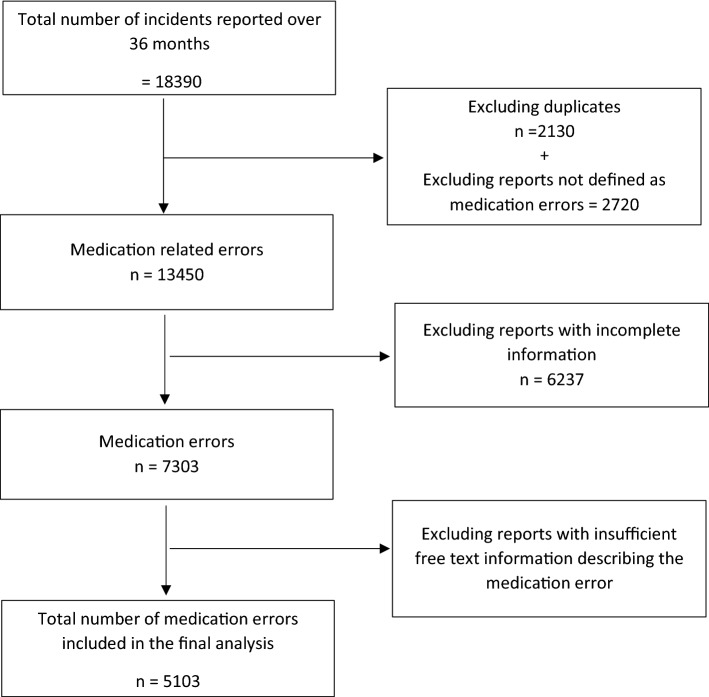
Inclusion and exclusion of medication errors for the final analysis

Of the 13,540 reports, 6237 were excluded from further analysis as having incomplete information (e.g. error type, severity) and a further 2200 with no or almost no free text description of the error. Only 5103 unique reports (37.7%) had sufficient information to be included in the remaining stages of analysis. Figure 2 is a flowchart of the fate of the medication error reports in this study.

**Fig. 3:**
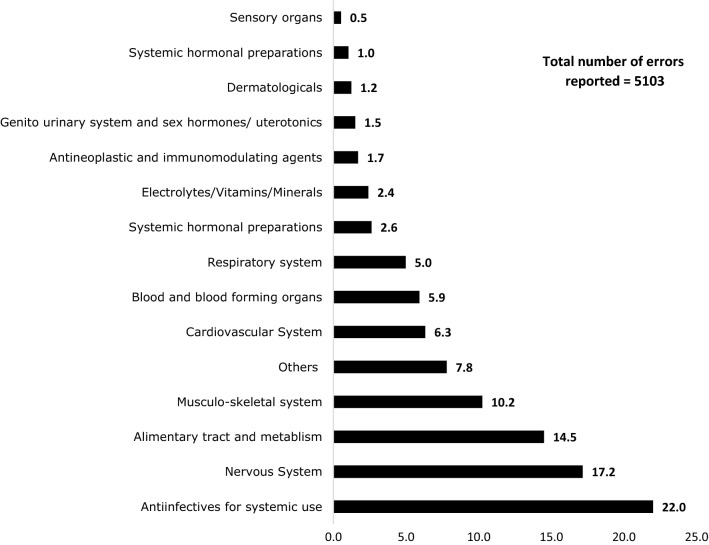
Drug categories associated with medication errors (%)

The majority of the reports (91.5%, n = 4667) were submitted by pharmacists followed by nurses (7.6%, n = 388) with very few (0.2%, n = 11) by doctors. Most reports (87.9%, n = 4485) were prescribing errors, with less administration errors (6.3%, n = 322), dispensing errors (5.1%, n = 260) and monitoring errors (0.7%, n = 36). The most common prescribing errors were wrong dose (36.1%, n = 1619), wrong frequency (14.6%, n = 658) and duplication (two or more medications from the same pharmacological grouping) (11.3%, n = 510). Medications were classified according to the Anatomical Therapeutic Chemical (ATC) classifications and anti-infectives for systemic use (22%, n = 1123) were the most common followed by medications used to treat nervous system disorders (17.2%, n = 876) (see Fig. 3).

According to the reporter, most reports (77.3%, n = 3,43) were either NCCMERP Category A (circumstances or events that have the capacity to cause error), followed by Category C (14.3%, n = 731) (an error occurred that reached the patient but did not cause patient harm), Category D (5.9%, n = 301) (an error occurred that reached the patient and required monitoring to confirm that it resulted in no harm to the patient and/or required intervention to preclude harm) and Category B (2.4%, n = 124) (an error occurred but the error did not reach the patient). Three errors (0.06%) were classified as Category E (wherein an error occurred and may have contributed to or resulted in temporary harm to the patient and required intervention) and only one error (0.02%) Category F (contributed to or resulted in temporary harm to the patient and required initial or prolonged hospitalization).

All 5104 error reports were analysed according Reason’s Accident Causality Model. Almost all (91.5%, n = 4671) were classified as active failures, of which comprised mistakes (66.1%, n = 3087), slips (16.6%, n = 774), lapses (12.7%, n = 593) and violations (4.6%, n = 217). The remainder were classified as error provoking conditions (8.4%, n = 429) and latent failure (0.04%, n = 2). The sub-classifications of these categories are given in Table 1. Note that many reports could not be classified due to incomplete information.

**Table 1:** Contributory factors based on Reason’s accident causation model, n = 5104

Active failures (mistakes), n = 3085	% (n)
Skill based mistakes	21.9 (675)
Knowledge based mistakes	4.0 (124)
Technology based mistakes	2.0 (62)
Others	2.0 (62)
Not enough information for classification	69.9 (2159)
Active failures (slips), n = 774	
Incomplete order	36.9 (286)
Selecting a wrong medication	35.6 (276)
Selecting a wrong dose	5.5 (43)
Wrong labeling	3.1 (24)
Look alike, sound alike medication	3.1 (24)
Others	8.0 (62)
Not enough information for classification	10.7 (83)
Active failures (lapses), n = 590	
Missing information (route/age/dose/weight etc.)	66.9 (395)
Omission	23.8 (141)
Failure to collect medication from pharmacy	2.0 (12)
Others	1.2 (7)
Not enough information for classification	6.1 (36)
Active failures (violations), n = 217	
Noncompliance (policy/procedure/orders)	94.0 (203)
Ordering contraindicated medication	3.2 (7)
Patient or caregiver	1.0 (2)
Others	1.3 (3)
Not enough information for classification	1.0 (2)
Error provoking conditions, n = 424	
Lack of knowledge	34.6 (148)
Reconciliation	17.9 (76)
Technology based errors (Cerner issues)	6.8 (29)
Communication problems	2.1 (9)
Environment factors	2.1 (9)
Others	25.8 (110)
Not enough information for classification	10.1 (43)
Latent factors, n = 2	
Organizational factors	100 (2)

## Discussion

The estimated incidence of medication errors in this study, as derived from medication error reports was 0.044%. Almost all reports were submitted by pharmacists for prescribing errors, which were largely wrong dose or wrong frequency errors. Most errors were considered by the reporter to be minor in nature. According to Reason’s Accident Causality Model [15], the vast majority were considered as active failures (slips, lapses, mistakes and violations).

Both NCCMERP and HMC have strategic aims that highlight the value of effective and efficient medication error reporting systems and practices in reducing error prevalence and severity [9, 18]. The findings of this study provide evidence that the reporting system and processes at HMC require review. Of the reports extracted, around one fifth were either duplicate reports or reports for incidents not classified as medication errors. Furthermore, of the remaining reports, just over one third had sufficient details to be included in the study. Of note, these reports can then not be used for the purpose of reflecting on healthcare practices hence will not contribute to improved patient safety. Several studies in other settings have also highlighted the issue of incomplete reports [22–24].

The incidence of medication errors, as identified in this study, is low compared to the figures reported in a recent systematic review wherein medication errors reported varied from 0.18 to 56 per 100 medication orders [10]. Various reasons could be attributed to these differences, most likely differences in error definitions, methodological approaches and outcome measures. Of the nine studies in the review which reported data derived from medication error reports, there was a lack of inconsistency in presentation of results [10]. Studies used terms of ‘errors per 1,000 admissions’, ‘errors per 100 prescriptions’, ‘errors per 1000 patient days’ etc., which limits any direct comparisons. There is a need to agree defined method and reporting standards for all such studies to facilitate data pooling, comparison and learning from best practice. Such developments would align to the aspirations of the WHO, ‘Medication Without Harm’ [1], and also provide a standardised benchmark for determining the impact of any interventions.

There are other complications to the interpretation of incidence data, which are likely to compromise its validity. To be valid, all medication errors have to be identified and promptly reported. There is accumulated evidence of widespread and significant under-reporting of medication errors by healthcare profession [25–32]. A recent mixed methods study conducted in Qatar identified a number of barriers to error reporting including fear and worry,that submitting was likely to lead to further investigation,concerns over the impact on working relationships,and the potential lack of confidentiality [16]. The incidence data derived from this study can therefore only be considered an estimate of the true incidence of medication errors in HMC. It is notable that almost all medication error reports were submitted by pharmacists, indicating potentially marked under-reporting by nurses and doctors.

While most errors were categorised as no harm, the severity rating was undertaken solely by the reporter hence may have been subjected to biases including reporting and social desirability. Rating the severity of medication errors is not straightforward hence the validity of these findings may be questionable. A systematic review of the tools used to assess prescribing error severity in studies reporting hospital prescribing error rates highlighted that 57% of 107 studies included in the review had an assessment of severity. While 40 different tools were identified, only two were considered to have acceptable reliability and validity [33]. While it may be useful for the reporter in HMC to consider the severity and consequences of the error, the potential validity issues should be borne in mind.

One strength of this study was the application of Reason’s Accident Causality Model in analysing the narrative description of the reports. While the findings will be dependent on the richness of the narrative (and in many instances this was incomplete), this does provide some indication of causality. Almost all errors were considered to be active failures (slips, lapses, mistakes and violations). The systematic review of medication errors in hospitals in the Middle East synthesised findings according to Reason’s Accident Causation Model, with similar results of active failures of slips, lapses and mistakes being most common [10]. Similar findings have been reported in systematic reviews of studies not restricted to the Middle East. In a review of prescribing errors in hospitalised patients, Tully et al. reported that the active failures were most frequently cited [34], as did Keers et al. in a systematic review of medication administration error studies [35].

This accumulation of evidence around active failures will be useful in considering any potential interventions aiming to reduce these factors. One limitation of Reason’s model is that it does not describe the full range of behavioural determinants potentially leading to errors occurring. While the research team had considered applying a behavioural change theoretical framework to characterize behavioural determinants, this could not be undertaken due to the lack of detailed information contained within the reports. The development of standardized reporting criteria to include these potential behavioural determinants would facilitate the development of targeted interventions aiming to reduce medication errors.

This study has several strengths including analysis of data collected over a three year period and the application of a theoretical framework within the study. There are, however, a number of study weaknesses hence the findings should be interpreted with caution. The study findings are dependent on data recorded in the error reports by the individual reporter, which are potentially subject to reporter bias by either under-reporting or selective reporting. Furthermore, as the study was conducted within HMC, the findings may not be generalisable to either settings within Qatar or beyond.

## Conclusion

While the medication error incidence rate in this study is lower than many other studies, almost all reports were submitted by pharmacists, indicating likely under-reporting. Effort is required to increase the effectiveness and efficiency of the reporting system. The use of the theoretical framework allowed identification of potential causes in relation to active failures, which can form the basis of interventions to improve medication safety.
